# Matrix metalloproteinase 1 1 G/2 G gene polymorphism is associated with acquired atrioventricular block via linking a higher serum protein level

**DOI:** 10.1038/s41598-020-66896-9

**Published:** 2020-06-18

**Authors:** Jan-Yow Chen, Kuan-Cheng Chang, Ying-Ming Liou

**Affiliations:** 1 Division of Cardiovascular Medicine, Department of Medicine, China Medical University, Taichung, Taiwan; 2 Department of Life Sciences, National Chung Hsing University, Taichung, Taiwan; 3 School of Medicine, China Medical University, Taichung, Taiwan; 4 The iEGG and Animal Biotechnology Center, and Rong Hsing Research Center for Translational Medicine, National Chung Hsing University, Taichung, Taiwan

**Keywords:** Cardiology, Clinical genetics

## Abstract

Limited studies are available regarding the pathophysiological mechanism of acquired atrioventricular block (AVB). Matrix metalloproteinases (MMPs) and angiotensin-converting enzyme (ACE) have been implicated in the pathogenesis of arrhythmia. However, the relationship between these molecules and acquired AVB is still unclear. One hundred and two patients with documented acquired AVB and 100 controls were studied. Gene polymorphisms of the MMP1 and ACE encoding genes were screened by the gene sequencing method or polymerase chain reaction-fragment length polymorphism assay, followed by an association study. The frequencies of the MMP1 −1607 2G2G genotype and MMP1 −1607 2 G allele were significantly higher in the AVB group than that in the controls (OR = 1.933, P = 0.027 and OR = 1.684, P = 0.012, respectively). Consistently, the level of serum MMP1 was significantly greater in acquired AVB patients than that in controls (6568.9 ± 5748.6 pg/ml vs. 4730.5 ± 3377.1 pg/ml, P = 0.019). In addition, the MMP1 2G2G genotype showed a higher MMP-1 serum level than the other genotypes (1G1G/1G2G) (7048.1 ± 5683.0 pg/ml vs. 5072.4 ± 4267.6 pg/ml, P = 0.042). MMP1 1 G/2 G gene polymorphism may contribute to determining the disease susceptibility of acquired AVB by linking the MMP serum protein level.

## Introduction

Atrioventricular block (AVB) is a group of abnormal AV conduction disorders caused by a delay or interruption in the impulse connection from the atria to the ventricles due to structural or functional impairment^1,2^. AVB is one of the major disorders for patients requiring pacemaker implantation^1,2^. In an AV block condition, the wavelets cannot reach the ventricles or are delayed through the AV node, slowing down the heart beat and causing a serious condition^1–3^. The main causes of acquired AVB include idiopathic fibrosis, aging process in cardiomyocytes, degeneration, ischemia, infarction, infiltration disease or drugs^1–4^. Progressive idiopathic fibrosis related to an aging process of the cardiac skeleton is the most common cause of chronic acquired AVB^2,4–8^_._ It is generally accepted that familial and inherited AVB are caused by gene mutations and dysfunction of the conduction system during heart development^9–12^. In contrast, the pathophysiological mechanism of acquired AVB is currently unclear.

Matrix metalloproteinases (MMPs) and renin-angiotensin system (RAS) acting agents, including the angiotensin-converting enzyme (ACE) and angiotensinogen, have been reported to promote tissue fibrosis and the aging process^13–15^. MMPs and extracellular matrix (ECM) have been shown to control cardiac conduction and have been linked to the modification of gap junction proteins for atrioventricular conduction^15,16^. Although the aging process and progressive fibrosis have been thought to be the key events for age-related conduction disturbances, ECM alteration was shown to cause impulse-conduction disorders^15–18^. Studies have shown that MMPs and RAS acting agents are involved in the pathogenesis of arrhythmia and cardiac conduction^17,19–23^. Studies in humans and animals demonstrated that modulation of MMP and RAS gene expression could lead to atrial fibrillation^19,20^, non-familial sick sinus syndrome^21^, and affect cardiac conduction after myocardial infarction^17^, apoptosis of the conductive system^22^ and occurrence of ventricular arrhythmia^23^. Here, we examined whether the polymorphisms of the genes encoding the MMPs and ACE could alter the disease susceptibility for acquired AVB by investigating the relationship between MMPs and ACE gene polymorphisms and disease susceptibility of acquired AVB. The results obtained indicated that MMP1 1 G/2 G gene polymorphism is associated with AVB via linking a higher serum protein level.

## Methods

### Study population

During the period from Sep 1, 2018 to Aug 31, 2019, a total of 102 patients with documented acquired AVB were enrolled as the patient group, and another 100 age and sex frequency-matched healthy individuals were enrolled as a control group (Table 1). The AVB was recorded and diagnosed with evidence of AV block by a series of electrocardiograms (ECG) and ambulatory ECG according to the international standard guidelines^24^. The criteria for inclusion were symptomatic second and third degree AVB, which met the indications for permanent pacemaker implantation. Patients with a history of familial AVB, severe systemic disease, acute coronary syndrome, drug-induced AVB, or AVB with reversible cause were excluded from this study. The control subjects free of AVB were randomly selected from the cardiovascular outpatient department and ward, and they were frequency-matched to AVB patients by gender and age at the China Medical University Hospital in Taiwan. The protocol of present study was approved by the Institutional Review Board of China Medical University Hospital in Taiwan (CMUH108-REC3-031). Written informed consent was obtained from each participant after a full explanation for the present study. The investigation conformed to the principles outlined in the Declaration of Helsinki. Here, we presented the data obtained according to the standard guideline, Strengthening the Reporting of Observational Studies in Epidemiology (STROBE), to improve the quality of this study^25^.

**Table 1:** General characteristics of patients included in the present study.

	AVB (N = 102)	Control (N = 100)	P
Age (years)	69.9 ± 14.8	69.6 ± 8.8	0.869*
Gender (male/female)	40/62	37/63	0.773^§^
BMI (kg/m^2^)	24.5 ± 3.7	25.1 ± 3.1	0.196*
HT (n, %)	45 (44.1%)	47 (47.0%)	0.681^§^
DM (n, %)	24 (23.5%)	20 (20.0%)	0.543^§^
CAD (n, %)	9 (8.8%)	13 (13.0%)	0.341^§^
AF (n, %)	6 (5.9%)	5 (5.0%)	0.782^§^

*T-test; ^§^χ^2^ test; AVB, atrioventricular block; BMI, body mass index; HT, hypertension; DM, diabetes mellitus; CAD, coronary artery disease; AF, atrial fibrillation.

## Genomic study

### Polymerase chain reaction

Blood samples from patients were prepared, and genomic DNA was isolated using a DNA extraction kit (ILLUSTRA, GE HEALTHCARE). Polymerase chain reaction (PCR) was performed with 100 ng genomic DNA, 2–6 pmol of selected primers, 1X TAG polymerase buffer, and 0.25 units of AMPLITAG GOLD polymerase (THERMO FISHER) in a final reaction volume of 50 μL using a programmable thermal cycler (GENEAMP PCR System 2700, APPLIED BIOSYSTEMS, CA). The polymorphisms for MMP genes selected for screening included MMP-1–320 T/C (rs494379), −340 T/C (rs514921), −422 T/A (rs475007), −519 A/G (rs1144393) and −1607 1 G/2 G (rs1799750), while the polymorphisms for ACE genes selected included ACE insertion/deletion (I/D) polymorphism.

### Genotyping

**Figure 1:**
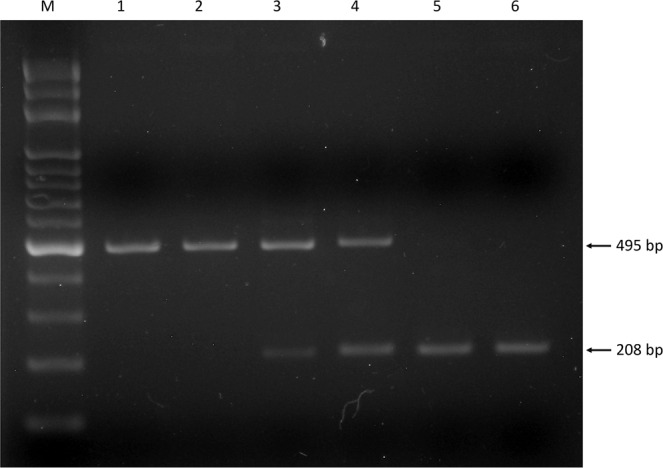
Electrophoresis for ACE I/D gene polymorphism. M represents the 100-base pair (bp) ladder. Lane 1 and 2 represent PCR products with 495-bp for homozygous genotype II, lanes 3 and 4 products with 495-bp and 208-bp for heterozygous genotype ID and lanes 5 and 6 products with 208 bp homozygous DD genotypes.

The genotype analysis of the subjects was studied by electrophoresis, polymerase chain reaction-fragment length polymorphism assay or direct gene sequencing if appropriate. Genotyping for the ACE I/D polymorphisms was studied by polymerase chain reaction-fragment length polymorphism assay. The primers for the ACE I/D gene polymorphisms were 5-TGGAGACCACTCCCATCCTTTCT-3 (forward) and 5-CAGGTCTTCATATTTCCGATGTGG-3 (reverse). Two fragments with different PCR product lengths were presented for the different genotypes (ACE I or ACE D genotypes). Electrophoresis was performed for genotyping. Thereafter, 10 µL of the products were loaded into a 3% agarose gel with ethidium bromide staining and separated by electrophoresis. Electrophoresis of the amplified products allowed to identify a 495-base pair fragment (insertion) and a 208-base pair fragment (deletion), respectively, on the gel for genotyping the ACE I/D polymorphisms. The gene polymorphisms were categorized as homozygotes (insertion type, II, as on lanes 1 and s), homozygotes (deletion type, DD, as on lanes 5 and 6), and heterozygotes (ID, as on lanes 3 and 4) as shown in Fig. 1. Genotyping for part of the PCR products was performed by gene sequencing for MMP1 gene polymorphisms. The primers for MMP1–320 T/C, −340 T/C, −422 T/A and −519 A/G gene polymorphisms were 5-TACAGGTGCATGACTCCATGCTTG-3 (forward) and 5-TCTAGAGTCGCTGGGAAGCTGTGA-3 (reverse). The primers for MMP1-1607 1 G/2 G gene polymorphisms were 5-ACATTGCAGGATGTGCAGGCTCTT-3 (forward) and 5-CTTGGGTACTGGTGACCGGTGTCA-3 (reverse). The gene sequences of the PCR products were subsequently determined by gene sequencing using a genetic analyzer (ABI 3730 XL DNA Analyzer, APPLIED BIOSYSTEMS).

### Association study

An association study between gene polymorphisms and AVB was performed to measure and compare the genotype distribution and allele frequency of the candidate genes in AV block and control groups.

### Measurement of serum MMP concentrations

Serum samples were randomly obtained from 75 acquired AV block patients and 74 control individuals. The samples were stored at −20 °C until the assays were performed. The serum MMP levels were detected using the enzyme-linked immunosorbent assay kit (ELISA) (INVITROGEN, CA, USA). The optical density (OD) was measured by an ELISA microplate reader. The concentration of MMP was determined from a standard curve according to the manufacturer’s instructions.

### Statistical analysis

Student’s t test was used when continuous data were collected. Categorical data were compared by the conventional chi-square test if the observation numbers in all categories were larger than 5; otherwise, the Fisher exact test was used. The genotype proportions for Hardy–Weinberg equilibrium (HWE) were assessed for each gene polymorphism by using the conventional goodness-of-fit test. Haplotype analysis for the gene polymorphisms was estimated by using HAPLOVIEW software^26^. Owing to the short distances between each polymorphism location on the MMP1 gene, gene polymorphisms may not separate by recombination and were in linkage disequilibrium (LD)^27^. Therefore, pairwise measurement of LD was performed to test the LD between the MMP1 gene polymorphisms. LD was estimated by D’. We also selected the *r*^2^ value to confirm LD because the magnitude of D′ significantly depends on the sample size^26,28^. Expectation-maximization (EM)-based haplotype frequency estimation was performed to determine whether any specific haplotypes were associated with acquired AVB on the basis of previous reports^26–29^. The statistical significance of LD was defined as *r*^2^ > 1/3 and D′ > 0.7, as suggested by previous reports^26–29^. A P value < 0.05 was considered statistically significant.

## Results

### Patient characteristics

The clinical features of AVB patients and controls are summarized in Table 1. Those patients with a history of familial AVB, severe systemic disease, acute coronary syndrome, drug-induced AVB, or AVB with reversible cause were excluded. The baseline clinical characteristics, including age, gender, percentage of patients with hypertension, diabetes mellitus (DM), coronary artery disease (CAD) and atrial fibrillation (AF), were similar between the 2 groups.

### HWE and LD measurements

**Figure 2:**
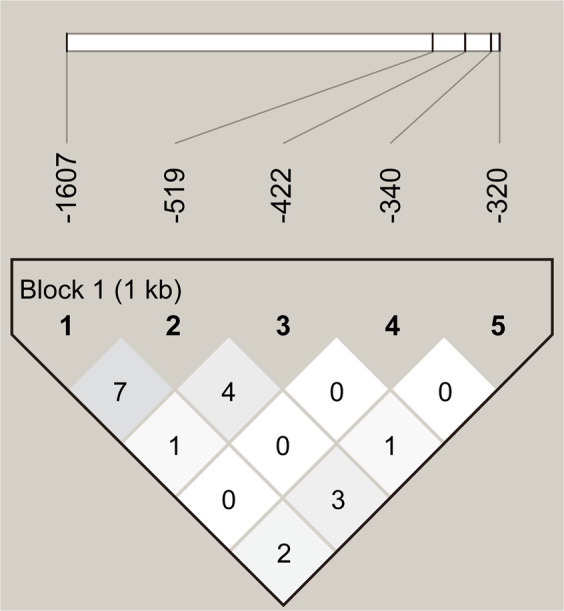
Linkage disequilibrium plot of MMP1 promoter loci. Pairwise linkage disequilibrium analysis shows *r*^2^ (x 100) values. The intensity of gray is proportional to *r*^2^.

Five polymorphic sites at positions −320, −340, −422, −519 and −1607 were found within the promoter region of the MMP-1 gene. The HWE for genotype distributions was assessed for each MMP1 promoter polymorphism at the −320, −340, − 422, −519 and −1607 positions for the AVB and control groups by the conventional chi-square goodness-of-fit test. The MMP1 genotype distribution in the AVB patients and controls did not significantly deviate from the HWE for the AVB patients (P-values were 0.724, 0.5952, 0.1116, 0.5434 and 0.1621, respectively) and control groups (P-values were 0.934, 0.838, 0.08, 1.0 and 0.2327, respectively). The pairwise linkage among these five polymorphic sites on the MMP1 promoter gene was evaluated by the LD test using D’ and r^2^. The D’ values of the loci pairs for −320/−340, −320/−422, −320/−519, −320/−1607, −340/−422, −340/−519, −340/−1607, −422/−519, −422/−1607 and −519/−1607 were 0.129, 0.137, 0.536, 0.265, 0.054, 0.758, 0.116, 1.0, 0.215, and 0.743, respectively. The corresponding values for *r*^2^ were 0.004, 0.012, 0.036, 0.031, 0.0, 0.009, 0.004, 0.045, 0.013, 0.085, respectively (Fig. 2). The D’ values indicated a significant linkage in the loci pairs of −340/−519 and −519/−1607. However, the *r*^2^ values for these three loci pairs were low and indicated no significant linkage in the loci pairs. This inconsistent result between the D’ and *r*^2^ values may be due to the relatively small sample size in this study^23–26^. The high D’ values and the low *r*^2^ values of these 2 loci pairs (−340/−519, −159/−1607) indicate an incomplete linkage among these 2 loci pairs, which explains the wide distribution of haplotypes for the MMP-1 gene.

ACE I/D gene polymorphisms were found for the ACE gene. The ACE I/D genotype distribution in AVB patients and the control group did not significantly deviate from HWE (P = 0.32 and 0.70, respectively).

### Single locus gene association analysis for MMP-1 gene polymorphisms and AVB

Five MMP1 gene polymorphisms (−1607 1 G/2 G, −519 A/G, −422 A/T, −340 C/T and −320 C/T) and one ACE gene polymorphism (I/D) were identified in our study. In addition, an association study showed that the ACE I/D polymorphisms were not associated with acquired AVB. A significant difference was noted in the distribution of the genotypes of MMP1 −1607 1 G/2 G polymorphisms between AVB and control subjects (P = 0.027). The 2G-dominant genotype (2G2G/1 G2G) frequency of the −1607 1 G/2 G polymorphism was significantly higher in acquired AVB patients than that in controls (OR = 2.59, 95% CI 1.014–6.587, P = 0.047). The 2G2G genotype frequency of the −1607 1 G/2 G2G polymorphism was significantly higher in the AVB group than that in the control group (45/102 vs. 29/100, OR = 1.933, 95% CI, 1.079–3.461, P = 0.027). The 2G allele frequency of −1607 1 G/2 G was significantly higher in the AVB group than that in the control group (140/204 vs. 113/200, OR = 1.684, CI 1.121–2.530, P = 0.012) (Table 2). The results of the single locus analysis indicated that the MMP1 −1607 1 G/2 G gene polymorphism is associated with the disease susceptibility of acquired AVB.

**Table 2:** Distribution of genotypes and alleles of MMP1 and ACE in patients with and without AV block.

Gene polymorphism	Genotypes and Alleles	AVB patients (N = 102)	Control patients (N = 100)	P
**MMP-1 gene**				
−1607 (1 G/2 G)	1G1G – n (%)	7 (6.9)	16 (16.0)	
	1G2G – n (%)	50 (49.0)	55 (55.0)	
	2G2G – n (%)	45 (44.1)	29 (29.0)	0.027
	2G2G/1G2G + 1G1G– n (%)	45 (44.1):57 (55.9) 29	29 (29.0):71(71.0)	0.027
	1G2G + 2G2G/1G1G – n (%)	95 (93.1):7 (6.9)	84 (84.0):16 (19.0)	0.047
	1G:2G– n (%)	64 (31.4):140 (68.6)	87 (43.5):113(56.5)	0.012
−519 (A/G)	**AA** – n (%)	88 (86.3)	81 (81.0)	
	AG– n (%)	14 (13.7)	18 (18.0)	
	GG – n (%)	0 (0)	1 (1.0)	0.389
	A:G – n (%)	190(94.1):14 (6.9)	180 (90.0):20 (10.0)	0.256
−422 (A/T)	**AA** – n (%)	47 (46.1)	52 (52.0)	
	AT– n (%)	39 (45.0)	35 (35.0)	
	TT – n (%)	16 (15.7)	13 (13.0)	0.692
	A:T – n (%)	133 (65.2):71(34.8)	139 (69.5):61(30.5)	0.356
−340 (T/C)	TT – n (%)	79 (77.5)	70 (70.0)	
	TC– n (%)	22 (21.6)	27 (27.0)	
	CC – n (%)	1 (1.0)	3 (3.0)	0.357
	T:C – n (%)	180 (88.2):24 (11.8)	167 (83.5):33 (16.5)	0.172
−320 (T/C)	TT – n (%)	19 (18.6)	17 (17.0)	
	TC– n (%)	52 (51.0)	48 (48.0)	
	TT – n (%)	31 (30.4)	35 (35.0)	0.803
	T:C – n (%)	90 (44.1):114 (55.9)	82 (41.0):118 (59.0)	0.526
**ACE gene**				
I/D	II – n (%)	51 (50)	46 (46.0)	
	ID – n (%)	40(39.2)	45 (45.0)	
	DD – n (%)	11 (10.8)	9 (9.0)	0.693
	**I**:D – n (%)	142(69.6): 62(30.4)	137 (68.5): 63(31.5)	0.81

AVB, atrioventricular block. *P* values obtained based on χ^2^ test or Fisher’s exact test; the upper *P* value is for comparison of genotype frequencies, and the lower is for allele frequencies.

### Association between the MMP1 promoter haplotypes and AVB

Seven major haplotypes in the MMP-1 promoter showing a haplotype frequency higher than 0.05 were identified in the present study, and their relationship with AVB was examined (Table 3). The 2G-A-T-T-T (−1607, −519, −420, −340, −320) haplotype demonstrated a significantly higher frequency in the AVB group than the control group (haplotype frequency: 0.1617 vs. 0.0875; OR = 2.01, P = 0.0239; Table 3). The results of the haplotype analysis showed a significant association between the MMP1 −1607 1 G/2 G gene polymorphism and AVB.

**Table 3:** Haplotype frequency estimates of the MMP-1 gene in patients with atrioventricular block and controls.

Haplotype					
−1607-519-420-340-320	Overall	AVB	Controls	OR	P
	(N = 202)	(N = 102)	(N = 100)		
2 G A A T C	0.221	0.2294 (46.8:157.2)	0.2130(42.6: 157.4)	1.10	0.6904
1 G A A T C	0.157	0.1392 (28.4 175.6)	0.1745 (34.9: 165.1)	0.76	0.3269
2 G A T T T	0.125	0.1617 (33.0: 177.0)	0.0875 (17.5:182.5)	2.01	0.0239
2 G A A T T	0.123	0.1363 (27.8:176.2)	0.1100 (22.0:178.0)	1.28	0.4160
1 G A T T C	0.067	0.0578 (11.8:192.2)	0.0760 (15.2:184.8)	0.75	0.4728
2 G A T T C	0.064	0.0784 (16.0:188.0)	0.0495 (9.9:190.1)	1.63	0.2358
1 G G A T T	0.054	0.0475 (9.7:194.3)	0.0595 (11.9:188.1)	0.79	0.5897

Haplotypes are not listed if all the estimated frequencies are <0.05 in patients with AVB, controls, and overall population.

### Serum MMP1 protein levels in the various MMP1 polymorphisms

**Figure 3:**
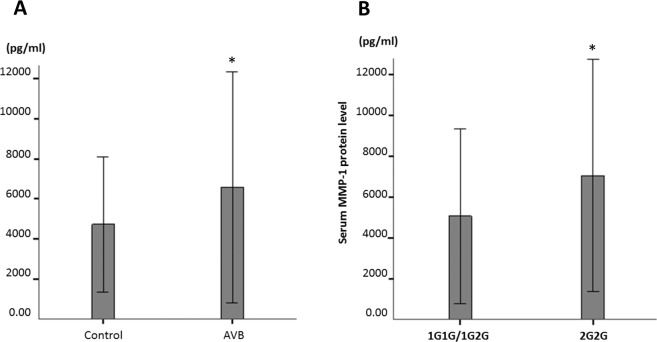
Serum MMP1 protein levels. (**A**) The serum MMP1 protein level in the AVB patients was significantly greater than that in the controls. (**B**) The serum MMP1 protein level of the studied subjects with a 2G2G genotype is significantly higher than that in subjects with non-2G2G genotypes in the present study. *<0.05.

The MMP1 serum protein level was significantly higher in the AV block group than in the control group (6568.9 ± 5748.6 pg/ml vs. 4730.5 ± 3377.1 pg/ml, P = 0.019). In addition, the 2G2G genotype showed a greater MMP-1 serum level than the 1G2G and 1G1G genotypes (7048.1 ± 5683.0 pg/ml vs. 5072.4 ± 4267.6 pg/ml, P = 0.042) (Fig. 3).

## Discussion

Increasing evidence suggests that gene mutations and dysfunction of the conduction system during heart development may result in familial AVB^9–12^. However, only a few data are available regarding the pathophysiology of acquired AVB. Idiopathic fibrosis is the most common cause of acquired AV block^5,6^. Since the possible pathogenesis of acquired AVB is quite different from familial AVB, it is of interest to explore the underlying mechanism of acquired AVB. In the present study, we found that the MMP1 1 G/2 G gene polymorphism is highly associated with acquired AVB via linking a higher serum MMP-1 level. The results of the present study may provide crucial and useful information for the prevention of acquired AVB.

### Single locus, haplotype analyses and serum level measurement of MMP1 gene polymorphisms

In a single locus analysis study, the results showed that the frequency of the MMP1 −1607 2 G2G genotype was significantly higher in the AV block group than that in the control group. The 2G-dominant (2G2G/2G1G) genotype frequency of the MMP-1 −1607 1G/2 G gene polymorphism was also significantly higher in the AV block group than in the controls. In the haplotype analysis, we found 7 major haplotypes of the MMP1 promoter polymorphism in the studied population (Table 3). The 2G-A-T-T-T haplotype was associated with a significant risk for AV block in comparison to the controls. These results suggested that the −1607 1 G/2 G polymorphism is a locus significantly associated with acquired AV block.

Consistently, the MMP1 serum protein level was significantly higher in the 2G2G genotypes than that in the other genotypes in the study population. The serum MMP1 protein levels were also higher in AVB patients than in the controls. These data suggested that MMP1 1 G/2 G gene polymorphism is associated with disease susceptibility for acquired AVB via linking a higher serum MMP1 protein level. Taken together, the results implicated that gene polymorphisms of MMP molecules are associated with the disease susceptibility of acquired AVB via linking the MMP1 serum protein levels.

### The role of MMP in tissue fibrosis and cardiac conduction

AVB has been reported to be closely related to fibrosis over the conduction system^5,6^. Tissue fibrosis is associated with MMP molecule expression, the renin-angiotensin system and TGF beta^13–15^. Studies using transgenic mice demonstrated that the overexpression of the angiotensin II type I (AT1) receptor in the myocardium is associated with AVB^30^. In a post MI rat model, downregulation of MMP has been demonstrated to protect disturbance in cardiac conduction^31^. A study of MMP deletion in a transgenic mouse model showed that MMP deletion decreased gap junction protein degradation and recovered cardiac conduction velocity in post-myocardial infarction mice^17^. Apparently, MMP plays an important role in the modification of cardiac conduction.

### The possible pathophysiology of MMP1 1 G/2 G gene polymorphisms affecting serum protein levels

The 1 G/2 G polymorphism has been reported to affect an Ets binding site and increase MMP1 gene transcription^32,33^. Consistently, a close association was found between the −1607 1 G/2 G polymorphism and MMP1 serum levels^34^. The MMP1 − 1607 2 G allele, but not the 1 G allele, accounted for the increased levels of pre-mRNA and protein. In addition, variation in MMP1 haplotypes has been reported to affect serum MMP-1 levels^34,35^. A study of the secondary structures of pre-mRNA of the MMP1 −1607 1 G/2 G gene polymorphism has shown that the pre-mRNA secondary structure of the 2 G allele is more stable than the 1 G structure based on deduction of the minimum free energy^34^. The lower free energy and more stable pre-mRNA structure of the 2 G allele may cause the increased expression of the 2 G/2 G gene polymorphism with higher serum protein levels and therefore increased the risk of acquired AV block^34^.

### Meta-analysis of genetic association study in diseases

A single case-control genetic study is of limited to reveal the true association between the genotypes and the disease susceptibility. Alternatively, meta-analysis is a useful statistical approach method to analyze the divergence among several independent studies by interrogating available data published. In addition, meta-analysis of genetic association study provide a better power to derive a more precise relationship between gene polymorphisms and disease susceptibility^36–38^. Currently, most of meta-analyses of genetic association studies are usually conducted by comparing genotype frequencies between cases and controls under various genetic models. A study with meta-analysis of genetic association has been conducted to show the relationship between MMP gene polymorphisms and lung cancer by logistic regression analysis^36^. In addition, meta-analysis has also been used to investigate the relationship of steroid medication and outcome of congenital conduction disorder^38^. However, no meta-analysis of genetic association study has been reported for the relationship between acquired AVB and MMP gene polymorphisms. To our best knowledge, the present study is the first case-control genetic association study to demonstrate that there is a close association between MMP1 gene polymorphisms and acquired AVB and provide functional results to clarify the underlying mechanism of acquired AVB. Our results may provide biological insights for acquired AVB. Thus far no other case-control genetic association study is available for resolving the relationship between MMP1 gene polymorphisms and the acquired AVB. The future study with a well-designed meta-analysis is needed to reconfirm the role of MMP gene polymorphisms in the disease susceptibility to acquired AVB.

### Study limitations

In the present study, we provided evidence obtained with the gene association study and with the functional measurement of serum MMP1 protein levels to support the crucial role of MMP1 gene polymorphism in the pathophysiology of acquired AVB. However, the direct impact of MMP1 gene polymorphism on cardiac cells located at the AV node still warrants further study. Another limitation is that the population of the present study is relatively small, and it needs to be further confirmed by large-scale studies.

## Conclusions

Patients with acquired AVB have a higher frequency of the 2 G allele and a higher distribution of 2 G2G genotype or 2G-dominant genotypes for the MMP1 1 G/2 G polymorphism. Consistently, the MMP1 serum protein level was significantly higher in the 2G2G genotypes than in the other genotypes in the study population. Taken together, these results suggest that the MMP1 1 G/2 G polymorphism is associated with the disease susceptibility of acquired AVB via linking higher serum MMP1 protein levels. Here, we provide the potential mechanism for the acquired AV block, which might be useful for disease prevention.
